# Data related to cyclic deformation and fatigue behavior of direct laser deposited Ti–6Al–4V with and without heat treatment

**DOI:** 10.1016/j.dib.2016.01.059

**Published:** 2016-02-04

**Authors:** Amanda J. Sterling, Brian Torries, Nima Shamsaei, Scott M. Thompson

**Affiliations:** a Department of Mechanical Engineering, Mississippi State University, Box 9552, MS 39762, United States; b Center for Advanced Vehicular Systems (CAVS), Mississippi State University, Box 5405, MS 39762, United States

**Keywords:** Fatigue, Cyclic deformation, Additive manufacturing, Laser Engineered Net Shaping (LENS), Ti–6Al–4V, Titanium

## Abstract

Data is presented describing the strain-controlled, fully-reversed uniaxial cyclic deformation and fatigue behavior of Ti–6Al–4V specimens additively manufactured via Laser Engineered Net Shaping (LENS) – a Direct Laser Deposition (DLD) process. The data was collected by performing multiple fatigue tests on specimens with various microstructural states/conditions, i.e. in their ‘as-built’, annealed (below the beta transus temperature), or heat treated (above the beta transus temperature) condition. Such data aids in characterizing the mechanical integrity and fatigue resistance of DLD parts. Data presented herein also allows for elucidating the strong microstructure coupling of the fatigue behavior of DLD Ti–6Al–4V, as the data trends were found to vary with material condition (i.e. as-built, annealed or heat treated) [1]. This data is of interest to the additive manufacturing and fatigue scientific communities, as well as the aerospace and biomedical industries, since additively-manufactured parts cannot be reliably deployed for public use, until their mechanical properties are understood with high certainty.

## Specifications Table

**Table:** 

Subject area	Engineering
More specific subject area	Fatigue, Mechanical Behavior, Additive Manufacturing
Type of data	Table (Microsoft Excel file format)
How data was acquired	Strain-controlled fatigue experiments (laboratory)
Data format	Raw and analyzed
Experimental factors	As-Built Samples: No pretreatment.
Annealed Samples: Soak at 704 °C for one hour in a muffle furnace, free convection cooling to room temperature
Heat Treated Samples: Soak at 1050 °C for two hours in an argon-purged tube furnace, furnace cool to room temperature
Experimental features	All samples were additively manufactured via Laser Engineered Net Shaping (LENS) using spherical Ti-6Al-4V powder consisting of a mesh size of −100/+325 (SAE AMS 4998C). The rods were machined into fatigue specimens following ASTM E606-9 [2]. All gage sections were polished to reduce surface effects. Fatigue testing was strain-controlled, and a thin layer of epoxy was placed between the gage section surface and the extensometer tines to prevent damaging the specimen surface.
Data source location	Center for Advanced Vehicular Systems (CAVS), Mississippi State University, Box 5405, Mississippi State, MS, USA
Data accessibility	The data is within this article.

## Value of the data

• Additive manufacturing provides many opportunities for generating efficient, conformal and customized parts. However, the ‘trustworthiness’ of these parts is still not well understood. Raw data demonstrating the mechanical behavior of these parts provides the scientific community the ability to further characterize their durability using various data interpretations.
• The material inspected, Ti–6Al–4V, is widely used in the biomedical and aerospace industries. The data presented allows for the comparison of components, such as medical implants or aircraft parts, manufactured via traditional methods with customized components manufactured via the LENS additive manufacturing technique.
• As most parts in applications undergo cyclic loading, fatigue is the most probable cause of failure. The data presented herein may be used for the design and mechanical characterization of LENS Ti–6Al–4V parts, which contain unique microstructures.
• Data presented herein can be used for calibrating and validating various analytical models and numerical simulations as they pertain to predicting the mechanical response of LENS Ti–6Al–4V.

## 1 Data

The data presented here consists of hysteresis and peak (maximum)/valley (minimum) stress–strain responses calculated via force and extensometer displacements recorded during fatigue testing of LENS Ti–6Al–4V specimens. Every effort was taken to ensure that standardized testing methods, as provided by ASTM E606/E606M-12 [2], were utlized. A summary of each test with corresponding strain amplitudes, test frequency, and reversals to failure are presented in Table 1, and can be acessed at the Data in Brief Dataverse: http://dx.doi.org/10.7910/DVN/7PPEDC.

## 2 Experimental design, materials and methods

All samples were fabricated as cylinders in the vertical direction using an OPTOMEC LENS 750 system equipped with a 1 kW Nd:YAG laser. Specimens were built using the following process parameters (all approximate): powder flow rate of 0.16 g/s, a traverse speed of 16.93 mm/s, and a laser power of 350 W. The build path of the samples consisted of a perimeter deposition at the start of each layer, followed by a hatch fill. The hatch fill alternated direction within each layer. As each layer was added, the hatch fill orientation was rotated 90° with respect to the previous hatch fill direction. Bothe the hatch and layer spacing were 0.508 mm. The overall diameter and height of the specimens were 10.92 mm and 101.6 mm, respectively. Three groups of specimens were inspected. The first group of specimens consisted of samples left in their as-built state. The second group of specimens was subjected to annealing; accomplished by holding the specimens at 704 °C for one hour and then allowing them to cool to room temperature via free convection. The third group of specimens underwent a heat treatment; accomplished by holding the specimens at 1050 °C for two hours and then allowing the samples to cool to room temperature via furnace cooling (Table 1).

All samples were machined into ASTM-compliant fatigue specimens for strain-controlled fatigue tests [2], and the gage sections were polished. Specimens were subjected to fully-reversed, strain-controlled fatigue tests performed using a MTS 858 machine along with a MTS model 634.31F-25 extensometer, in compliance with ASTM E606/E606M-12 standard [2]. In order to produce similar strain rates between tests, a sinusoidal loading profile was utilized. The test frequency was adjusted for each tested strain level in order to avoid any frequency effects on the cyclic behavior. To prevent the sharp extensometer tines from damaging the gage section surface, a thin layer of epoxy was applied in those areas as a protective barrier [1].

## Figures and Tables

**Table 1:** Summary of fully-reversed (R =−1) strain controlled fatigue tests.

**Sample Designation**	**ε_a_ (%)**	**Frequency (Hz)**	**2N_f_ (Reversals)**
**As-built LENS**			
0.2%_7Hz	0.2	7	> 4,061,240
0.3%_7Hz (a)	0.3	7	3,375,860
0.3%_7Hz (b)	0.3	7	619,936
0.3%_7Hz (c)	0.3	7	491,592
0.35%_5Hz (a)	0.35	5	119,954
0.35%_5Hz (b)	0.35	5	100,646
0.4%_4Hz (a)	0.4	4	91,080
0.4%_4Hz (b)	0.4	4	90,218
0.5%_3Hz (a)	0.5	3	68,516
0.5%_3Hz (b)	0.5	3	64,410
0.7%_2Hz (a)	0.7	2	3,422
0.7%_2Hz (b)	0.7	2	2,728
1%_2Hz (a)	1.0	0.5	998
1%_2Hz (b)	1.0	0.5	746
**Annealed LENS**			
0.3%_5Hz (a)	0.3	5	210,334
0.3%_5Hz (b)	0.3	5	119,506
0.35%_2Hz (a)	0.35	2	166,322
0.35%_2Hz (b)	0.35	5	102,712
0.4%_2Hz	0.4	2	133,100
0.5%_3Hz (a)	0.5	3	25,616
0.5%_2Hz (b)	0.5	2	15,026
0.5%_3Hz (c)	0.5	3	12,464
0.7%_2Hz	0.7	2	5,266
**Heat Treated LENS**			
0.3%_7Hz	0.3	7	627,774
0.35%_5Hz (a)	0.35	5	922,610
0.35%_5Hz (b)	0.35	5	864,070
0.5%_3Hz (a)	0.5	3	74,054
0.5%_3Hz (b)	0.5	3	51,990
1%_0.5Hz (a)	1.0	0.5	538
1%_0.5Hz (a)	1.0	0.5	244
