# Supplementary material for: Data related to cyclic deformation and fatigue behavior of direct laser deposited Ti–6Al–4V with and without heat treatment
Source: Data Brief. 2016 Feb 4;6:970–3. doi: 10.1016/j.dib.2016.01.059 (PMC4758181; doi:10.1016/j.dib.2016.01.059)
Supplement: Supplementary file 1 — Supplementary material [file mmc1.doc]

**CONFLICT OF INTEREST DECLARATION**

We wish to confirm that there are no known conflicts of interest associated with this publication and there has been no significant financial support for this work that could have influenced its outcome.
