# Supplementary material for: Data related to cyclic deformation and fatigue behavior of direct laser deposited Ti–6Al–4V with and without heat treatment
Source: Data Brief. 2016 Feb 4;6:970–3. doi: 10.1016/j.dib.2016.01.059 (PMC4758181; doi:10.1016/j.dib.2016.01.059)
Supplement: Supplementary file 2 — Supplementary material [file mmc2.pdf]

Appendix A: Supporting Information

Accompanied data for each sample include two types of dataset: “peak valley” and “cyclic deformation”. The “peak valley” files contain peak (maximum) and valley (minimum) stress and strain measurements for each collected cycle in a logarithmic increment, the time at which these measurements were taken, and the number of reversals. The “cyclic deformation” files contain hysterisis stress and strain measurements recorded throughout the test, and the corresponding time at which each measurement was taken, and the number of reversals. The data in “cyclic deformation” files were originally recorded for each cycle in a logarithmic increment. However, these files have been trimmed to produce a manageable file size while still preserving general trends of stress-strain responses. In these files, the hysteresis stress-strain responses are presented in a 2^n increment for each recorded cycle up to the 100th cycle. After the 100th cycle, the data of every other recorded cycle are included until the failure of the specimen. It is to be noted that the specimen name used in this article and data files are consistent with the name presented in [1]. A summary of each test with corresponding strain amplitudes, test frequency, and reversals to failure are presented in Table 1. The present data includes:

- As Built LENS_Cyclic Deformation file: The document includes all cyclic fatigue data for the as-built LENS Ti-6Al-4V specimens.
- As Built LENS_Peak Valley file: The document includes the peak valley fatigue data for the as-built LENS Ti-6Al-4V specimens.
- Annealed LENS_Cyclic Deformation file: The document includes all cyclic fatigue data for the annealed LENS Ti-6Al-4V specimens.
- Annealed LENS_Peak Valley file: The document includes the peak valley fatigue data for the annealed LENS Ti-6Al-4V specimens.

- Heat Treated LENS_Cyclic Deformation file: The document includes all cyclic fatigue data for the heat treated LENS Ti-6Al-4V specimens.
- Heat Treated LENS_Peak Valley file: The document includes the peak valley fatigue data for the heat treated LENS Ti-6Al-4V specimens.
